# Oncocytoma of the Parotid Gland with Facial Nerve Paralysis

**DOI:** 10.1155/2018/7687951

**Published:** 2018-06-21

**Authors:** Seijiro Hamada, Keishi Fujiwara, Hiromitsu Hatakeyama, Akihiro Homma

**Affiliations:** ^1^Department of Otolaryngology Head and Neck Surgery, Faculty of Medicine and Graduate School of Medicine, Hokkaido University, N15W7, Kita-Ku, Sapporo 0608638, Japan; ^2^Department of Otolaryngology Head and Neck Surgery, Yokohama City University Medical Center, 4-57 Urafune-cho, Minami-ku, Yokohama 2320024, Japan

## Abstract

Parotid gland tumor with facial nerve paralysis is strongly suggestive of a malignant tumor. However, several case reports have documented benign tumors of the parotid gland with facial nerve paralysis. Here, we report a case of oncocytoma of the parotid gland with facial nerve paralysis. A 61-year-old male presented with pain in his right parotid gland. Physical examination demonstrated the presence of a right parotid gland tumor and ipsilateral facial nerve paralysis of House–Brackmann (HB) grade III. Due to the facial nerve paralysis, a malignant tumor of the parotid gland was suspected and right parotidectomy was performed. Oncocytoma was confirmed histopathologically. The facial nerve paralysis was resolved 2 months after surgery. During the follow-up period (one and a half years), no recurrence was observed. As the tumor showed a distinctive dumbbell shape and increased somewhat due to inflammation (i.e., infection), the facial nerve was pinched by the enlarged tumor. Ischemia and strangulation of the nerve were considered to be the cause of the facial nerve paralysis associated with the benign tumor in this case.

## 1. Introduction

Parotid tumor with facial nerve paralysis is generally considered as a criterion for malignancy. However, several case reports have documented benign tumors of the parotid gland with facial nerve paralysis, with almost all of these cases diagnosed as Warthin's tumor [1–5] or pleomorphic adenoma [6, 7]. Oncocytomas are rare benign tumors that comprise about 0.1 to 1.5% of all salivary gland tumors [8].

We herein describe the case of a 61-year-old male who suffered from right parotid oncocytoma with facial nerve paralysis.

## 2. Case Presentation

**Figure 1:**
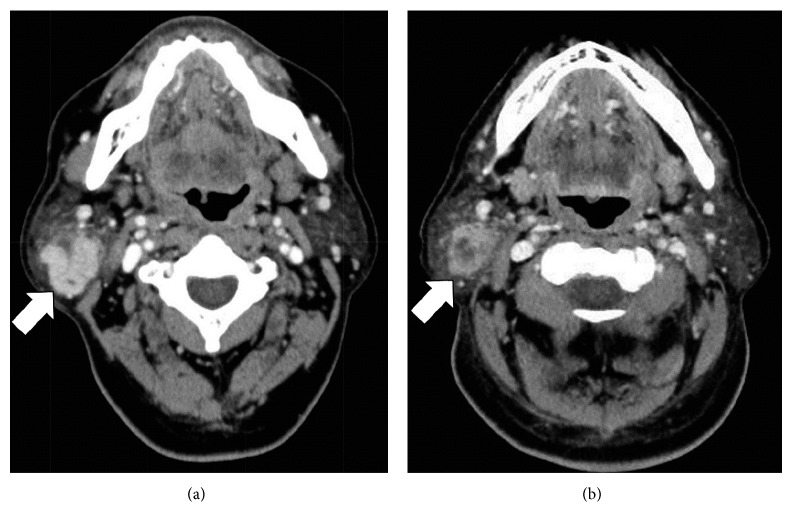
Postcontrast computed tomography (CT) findings. The right parotid gland tumor in scans obtained in our hospital (b) is smaller than that in scans obtained in our affiliated hospital (a).

**Figure 2:**
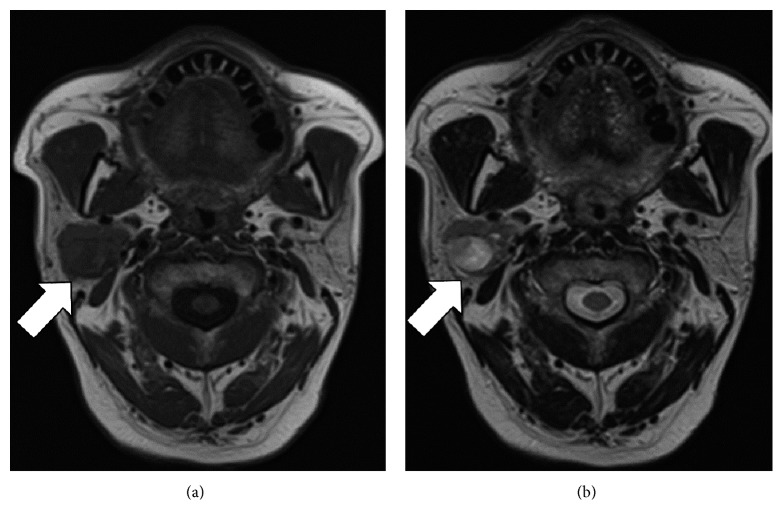
MR imaging findings. (a) A T1-weighted image showing the tumor to have lower intensity than that of the native parotid tissue. (b) A T2-weighted image showing the tumor to have an intermediate signal intensity and partial hyperintensity.

A 61-year-old male had been aware of a right parotid mass for about 10 years; however, he did not seek treatment as the mass was painless. On experiencing serious right parotid pain, he visited our affiliated hospital. Physical examination revealed a painful mass in his right parotid gland of approximately 30 mm in diameter and ipsilateral facial nerve palsy of House–Brackmann (HB) grade III. Laboratory findings showed a leukocyte count of 12,190/*μ*L and C-reactive protein (CRP) of 0.18 mg/dL. Computed tomography (CT) revealed an enhanced irregularly shaped mass in the right parotid gland (Figure 1(a)). T1-weighted SE MR imaging of the mass showed lower intensity than that of the native parotid tissue (Figure 2(a)). T2-weighted SE MR imaging also showed intermediate signal intensity and partial hyperintensity (Figure 2(b)). At this stage, a malignant neoplasm of the parotid gland was suspected.

Nine days after the appearance of symptoms, he was referred to our hospital. On physical examination, the mass was found to be still present but the pain had eased. In addition, the facial nerve palsy showed some improvement to HB grade II. Ultrasound examination revealed an inhomogeneous, lobulated mass (approximately 30 × 25 mm) in the right parotid gland. CT showed that the tumor had become smaller than at the time of the previous scan in our affiliated hospital (Figure 1(b)). We tried ultrasound-guided fine needle aspiration cytology (FNAC) twice, with an initial finding of necrotic material and a subsequent finding of a large number of histiocytes and acinar cells and a small number of eosinophilic cells with no atypical findings observed. However, no definitive diagnosis was provided.

We suspected a malignant tumor because of the associated facial nerve paralysis and parotid pain. On the contrary, we considered the possibility of a benign tumor with inflammation due to the reduction in tumor size and pain. We planned a total parotidectomy including exeresis and reconstruction of the facial nerve. However, we also made preparations to preserve the facial nerve should a benign tumor be suggested during the surgery.

**Figure 3:**
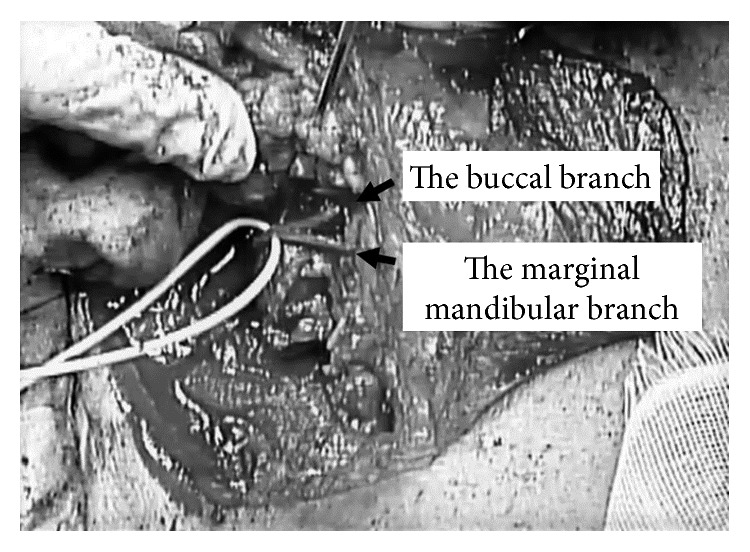
Intraoperative view of the facial nerve. The buccal branch and marginal mandibular branch can be seen to be compressed by the tumor.

The patient underwent surgery at 31 days after the appearance of symptoms. A modified Blair incision was made, and a dumbbell-shaped parotid tumor, extending from the superficial to the deep lobe of the parotid gland, was identified. The buccal and marginal mandibular branches of facial nerve were pinched by the tumor (Figure 3). The nerve branches were in contact with the tumor, but not involved. The temporal and zygomatic branches were present on the tumor capsule. A portion of the tumor was cut off and used for frozen section diagnosis (FSD). It was found to consist of solid and cystic components. The solid component was surrounded by a fibrous capsule, and the wall of the cyst component was lined by a stratified squamous epithelium, which did not show any atypical changes. Based on the above results, we considered the tumor to be definitely benign. We managed to keep the facial nerve away from the tumor by use of microscissors, and the tumor was removed between the zygomatic and buccal branches.

**Figure 4:**
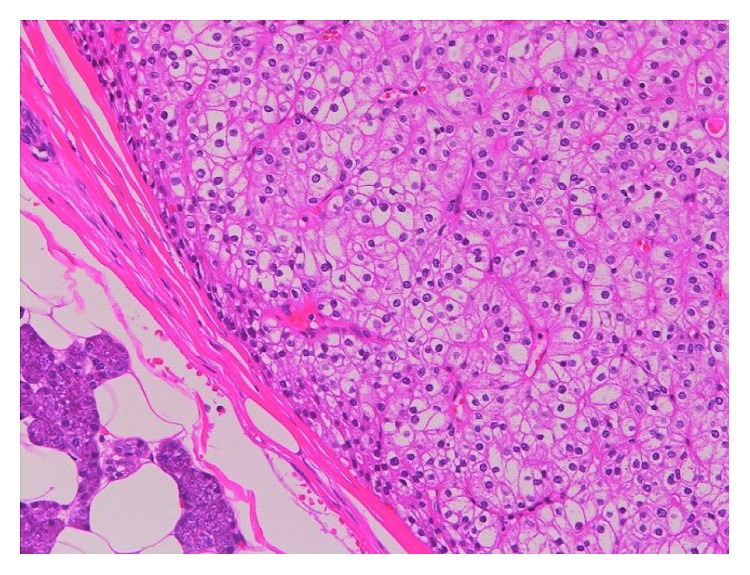
Frozen section of the neoplasm. Clear cells can be seen to form a clear boundary between the normal parotid gland tissues (haematoxylin-eosin, ×100).

Histopathological examination showed the tumor contained clear cells, which formed a clear boundary between the normal parotid gland tissues (Figure 4). The Ki-67 labelling index was exceedingly low and the mitotic count was negative. These findings were consistent with oncocytoma of the parotid gland.

Throughout the postoperative period, the nasalis muscle of the affected side was slightly weakened but it was completely recovered at 2 months after surgery. No recurrence was observed during the 18-month follow-up period.

## 3. Discussion

Oncocytomas are benign neoplasms composed of oncocytes: large cells with abundant granular and eosinophilic cytoplasm [9]. Oncocytomas are rare, comprising only about 0.1 to 1.5% of all salivary gland tumors [8]. The parotid gland is the most common site of oncocytic changes [10], but they have also been noted in the submaxillary gland, sublingual gland, larynx, soft palate, hard palate, and nasal cavities. They often present as solitary slow growing painless masses, which are smooth and with some mobility upon clinical examination. On postcontrast CT, around 50% of oncocytomas demonstrate inhomogeneous enhancement. Some oncocytomas consist of a curved nonenhanced lesion and cystic lesion, corresponding to central scar tissue and cystic degeneration, respectively. As the majority of oncocytomas appear as isointense in comparison to the parotid tissue on MRI with T2 and T1, oncocytomas are often called “vanishing tumors” [9]. However, some oncocytomas show different intensities. In fact, the tumor in our case showed lower intensity on T1-weighted and intermediate signal intensity and partial hyperintensity on T2-weighted images. The usefulness of diffusion-weighted MR imaging for differentiating between oncocytomas and Warthin's tumors has been reported [11]. In this case, we could not obtain accurate diagnosis of the tumor by FNAC. Retrospectively, it is difficult to diagnose oncocytoma based on the findings of FNAC due to only a small number of eosinophilic cells. It has been reported the sensitivity for the detection of oncocytoma by FNAC is 29% [12]. Eventually, surgical management with parotidectomy was essential for diagnosis and treatment. During histopathological examination, it is important to distinguish oncocytomas from oncocytic carcinomas. Oncocytic carcinomas are composed of malignant oncocytes with adenocarcinomatous architectural phenotypes and infiltrative qualities, including local invasion and regional or distant metastases [13]. Furthermore, the frequency of Ki-67-positive cells with nuclear staining was shown to be higher in oncocytic carcinomas than in oncocytomas [13]. These findings were not observed in this case.

Eneroth [14] reported that, in a series of 2,261 patients with parotid tumors, peripheral facial nerve paralysis developed spontaneously in 46 patients. These 46 patients all had malignant parotid tumors, on the basis of which he concluded that facial nerve paralysis must be considered as a criterion of malignancy. However, we encountered a benign parotid gland tumor with facial nerve paralysis. Similar cases have been reported in the literature. Histopathologically, Warthin's tumor is the most commonly reported [1–5], with pleomorphic adenoma [6, 7] and lipoma [15] also reported. Only 2 cases of oncocytoma with facial nerve paralysis have been reported to date [16, 17], with one patient showing painful swelling in the parotid area similarly to that observed in our case [16]. Therefore, our case is considered to be extremely rare. The causes of facial nerve paralysis with benign tumor reported in other studies include the facial nerve being compressed due to a sudden increase in tumor volume. For example, a histological examination of nerve bundles led to the speculation that ischemia of the nerve caused by external compression resulted in facial nerve palsy [3]. A case of spontaneous intratumoral hemorrhage in parotid oncocytoma was also reported [18]. Necrosis, inflammation, and fibrosis surrounding the branches of the facial nerve have also been considered to be a cause of palsy [1]. In our case, the patient suffered serious right parotid pain temporarily. When he visited our hospital, the pain had diminished and CT examination showed that the tumor had become smaller than at the time of the previous scan at our affiliated hospital. As the tumor showed a distinctive dumbbell shape and increased in size somewhat by inflammation (i.e., infection), the facial nerve was pinched by the enlarged tumor. Ischemia and strangulation of the nerve were considered as the reason for the facial nerve paralysis with benign tumor in this case.

## 4. Conclusion

We reported a parotid gland oncocytoma with facial nerve paralysis. We initially suspected a malignant tumor; however, we later suspected a benign tumor with inflammation based on the reduction in tumor size and pain. We, therefore, planned operative methods to cover either eventuality. As a result, we could resect the tumor while preserving the facial nerve.
